# Impact of early aggressive treatment on long-term biochemical marker patterns in inflammatory bowel disease

**DOI:** 10.1007/s00535-025-02244-w

**Published:** 2025-05-02

**Authors:** Yu Kyung Jun, Yonghoon Choi, Cheol Min Shin, Young Soo Park, Nayoung Kim, Dong Ho Lee, Soyeon Ahn, Hyuk Yoon

**Affiliations:** 1 Department of Internal Medicine, Seoul National University Bundang Hospital, Seongnam, South Korea; 2 Department of Internal Medicine and Liver Research Institute, Seoul National University College of Medicine, Seoul, South Korea; 3 Division of Statistics, Medical Research Collaborating Center, Seoul National University Bundang Hospital, Seongnam, South Korea

**Keywords:** Inflammatory Bowel Diseases, Crohn’s Disease, Latent Class Analysis

## Abstract

**Backgrounds:**

The disease course of inflammatory bowel disease (IBD) is highly variable; early and precise identification of patients with poor outcomes is crucial. We aimed to classify the long-term disease course of IBD using biochemical markers and evaluate the clinical factors associated with different disease courses.

**Methods:**

A latent class mixed model was employed to identify distinct trajectories of C-reactive protein (CRP) and fecal calprotectin (FCP) levels in 256 and 635 patients with Crohn’s disease (CD) and ulcerative colitis (UC), respectively, from a tertiary hospital cohort. Multinomial logistic regression was used to evaluate the relationships between various trajectories and clinical variables.

**Results:**

Three trajectories were identified for CD and UC: class 1, early and sustained biochemical remission; class 2, delayed remission; and class 3, prolonged difficulty in achieving remission for > 5 years. For patients with CD, early immunomodulator initiation was associated with a high likelihood of belonging to class 1 in the CRP trajectory analysis, whereas early advanced therapy increased the probability of belonging to class 1 in the FCP trajectory analysis. CRP trajectory analysis showed no significant associations in patients with UC. Younger age at diagnosis and early immunomodulator initiation were associated with higher odds of being in class 2 or 3, whereas current smoking was associated with a high likelihood of being in class 1 in the FCP trajectory analysis.

**Conclusions:**

Early aggressive medical treatment for CD may lead to long-term biochemical remission, whereas no similar association was observed in UC.

**Supplementary Information:**

The online version contains supplementary material available at 10.1007/s00535-025-02244-w.

## Introduction

Inflammatory bowel disease (IBD) is a chronic inflammatory condition of the gastrointestinal tract characterized by alternating periods of improvement and exacerbation, with marked heterogeneity in disease progression [1]. Some patients maintain clinical remission for extended periods; however, others experience severe colitis with complications that may necessitate bowel resection, result in permanent disability, or lead to colitis-associated colorectal cancer. Therefore, early identification of patients at risk of poor prognosis and the development of effective personalized treatment strategies based on risk levels are essential.

C-reactive protein (CRP) and fecal calprotectin (FCP) are crucial biochemical markers used to distinguish between patients with IBD and healthy individuals, as well as to assess disease activity in IBD [2, 3]. The Selecting Therapeutic Targets in IBD (STRIDE)-II guidelines set achieving biochemical remission as an intermediate goal for IBD treatment because biochemical remission is associated with better long-term outcomes compared with clinical remission [1, 4–7].

Previous studies have addressed temporal changes in IBD. In the Inflammatory Bowel South-Eastern Norway (IBSEN) cohort, clinical courses over 10 or 20 years from IBD diagnosis were categorized into four curves, with each curve representing a different pattern of symptom severity [8–10]. Patients in the IBSEN cohort selected the curve that best reflected changes in bowel symptoms over time [8–10]. However, this categorization is based on patient self-reports and lacks objective evidence. Additionally, because the IBSEN study included patients diagnosed between 1990 and 1993, it may not reflect the disease course in the current era of advanced therapies. Patients with Crohn’s disease (CD) were recently categorized into four groups based on longitudinal changes in FCP using latent class mixed models (LCMMs). Smoking at diagnosis and upper gastrointestinal involvement were associated with elevated FCP trajectories, whereas early biologic therapy was associated with downward FCP trajectories [11]. However, CRP trajectories were not analyzed, which is another biomarker emphasized in the STRIDE II cohort, and patients with ulcerative colitis (UC) were excluded. Based on previous studies [8–12], we used LCMMs to characterize patients with CD and UC based on longitudinal changes in CRP and FCP and investigated the correlation between each subclass and IBD characteristics.

## Materials and methods

### Patient population

This longitudinal study used data from patients who were prospectively enrolled in the IBD cohort at Seoul National University Bundang Hospital between April 2017 and November 2023. Data on patient demographics, comorbidities, disease characteristics, and disease course were collected every time the patient visited the clinic using the Research Electronic Data Capture program (REDCap; Vanderbilt University, Nashville, TN, USA), a secure web-based application that supports data capture for research [13]. Only patients who underwent CRP and FCP testing at least twice between 6 months and 5 years after IBD diagnosis were included. All CRP and FCP results obtained after IBD diagnosis were used in the latent class analysis (LCA). The study protocol was approved by the Institutional Review Board of Seoul National University Bundang Hospital (No. B-2310 - 859- 102).

### CRP and FCP measurements

Longitudinal data on CRP and FCP levels from 2017 to 2023 were collected. Patients in the IBD cohort routinely had their CRP and FCP levels measured during clinic visits. For CRP measurements, blood samples were collected using serum separating tubes and measured using a high-sensitive latex agglutination or immunoassay rate method. CRP measurements were performed using the Atellica CH Analyzer, Atellica CH C-Reactive Protein_2 kits (Siemens Healthineers, Erlangen, Germany), AU5800 analyzer, and CRP Latex kits (Beckman Coulter, Brea, CA, USA). For FCP measurements, 50–100 mg of stool samples were collected using a standard stool container and submitted to the laboratory within 24 h. FCP levels were measured using a fluorescence enzyme immunoassay with a Phadia 200 and EliA Calprotectin 2 Well (Thermo Fisher Scientific, Phadia, Sweden) [14]. The range for CRP was 0.01–30 mg/dL: results of > 30 mg/dL were set to 30 mg/dL and those of < 0.01 mg/dL to 0.01 mg/dL. The range for FCP was 3.8–6000 μg/g: results of > 6000 μg/g were set to 6000 μg/g and those of < 3.8 μg/g to 3.8 μg/g.

### Variables

Patient demographics, comorbidities, IBD characteristics, and treatment history were retrieved from the REDCap database. Smoking history, history of appendectomy, and other underlying diseases were obtained through patient surveys. The IBD characteristics were classified according to the Montreal classification [15]. An anti-neutrophil cytoplasmic antibody (ANCA) titer of ≥ 1:20 was considered positive [16]. The elevation of anti-*Saccharomyces cerevisiae* antibody (ASCA) immunoglobulin A (IgA) or IgG was defined according to a serum concentration of ASCA IgA or IgG ≥ 25 UA/mL [17].

Prescription information for corticosteroids (CS), immunomodulators (IMs: thiopurine, methotrexate), and advanced therapies (AT: biologics and small molecules) was recorded. ATs included anti-tumor necrosis factor (TNF) agents (infliximab, adalimumab, and golimumab), vedolizumab, ustekinumab, and tofacitinib [18–20]. Treatment within 6 months of IBD diagnosis was regarded as “early” treatment [21]. Early emergency room (ER) visits or hospitalizations were identified if patients visited the ER or required hospitalization within 6 months after IBD diagnosis because of symptom aggravation or complications from IBD. Early intestinal resection, performed within 6 months of CD diagnosis because of acute complications, such as intestinal obstruction, perforation, or intra-abdominal abscess, was also investigated in patients with CD [22].

### Statistical analysis

Data are presented as the mean ± standard deviation for continuous variables and as frequency (%) for categorical variables. LCA is a statistical technique used to identify unobserved (latent) subclasses within a population based on individual responses to multiple observed variables [23, 24]. LCMMs extend traditional LCA by integrating the features of mixed models, thereby enabling the handling of longitudinal or hierarchical data. This model allows the identification of latent subclasses while modeling random effects and time trends [25]. The LCMM approach was employed to identify homogeneous groups of patients with IBD according to the longitudinal trajectories of CRP or FCP levels during the follow-up period.

The CRP and FCP values were log-transformed to normalize the distributions and reduce the influence of outliers. In the LCMM, the long-term time trends of the CRP or FCP data were estimated using natural cubic splines ranging from one to six classes. The optimal number of classes was selected by comprehensively considering the trajectory plots, Akaike information criterion, and Bayesian information criterion in conjunction with clinical interpretation. An alluvial plot was created to illustrate how additional classes emerged as the number of assumed classes increased.

Finally, univariate analyses and multinomial logistic regression were used to assess differences in patient demographics and clinical factors according to latent CRP or FCP classes. The covariates evaluated in the univariate analyses included sex, age at diagnosis, smoking status, history of appendectomy, Charlson comorbidity index [26], ANCA titer, ASCA IgA/IgG, early medical treatment history (CS, IM, and AT), early ER visits, and early hospitalization. For CD, disease location, behavior, and early intestinal resection were included. Disease extent was also included as a covariate in the UC analyses. Frequencies were compared using the chi-square test, and mean values among the classes were compared using one-way analysis of variance. All variables that met the criteria for statistical significance in the univariate analyses (*p*-value < 0.2) were included in the multinomial logistic regression. In the multinomial logistic regression, a *p*-value < 0.05 was considered statistically significant. Univariate analyses and multinomial logistic regression were repeated for the sensitivity analysis, which only included patients with a high probability (> 70%) of being grouped into specific latent classes. R (version 4.3.0; R Foundation for Statistical Computing, Vienna, Austria) was used for all statistical analyses.

## Results

### Modeling of CRP and FCP trajectories

Among patients with CD, 245 and 256 were included in the CRP and FCP models, respectively (Supplementary Fig. 1). Among the patients with UC, 635 and 536 were included in the CRP and FCP models, respectively. The LCMMs fitted with two to six assumed classes achieved convergence based on the default convergence criteria (Supplementary Fig. 2). In all four models, the maximum log-likelihood and Akaike information criterion decreased with an increasing number of classifications, whereas the models with one class had the smallest Bayesian information criterion, except for the CRP model for UC. The performance metrics for each model are presented in Supplementary Table 1. We concluded that using a three-class assignment provided the most consistent analysis despite variations in the patient groups (CD and UC) and models (CRP and FCP). The median intervals between FCP measurements were approximately 2.5 months in patients with CD and 3 months in those with UC, respectively. The median interval between CRP measurements was approximately 1.5 months in patients with CD and UC (Supplementary Fig. 3). No notable differences were observed in the intervals for FCP and CRP measurements across classes.

**Fig. 1:**
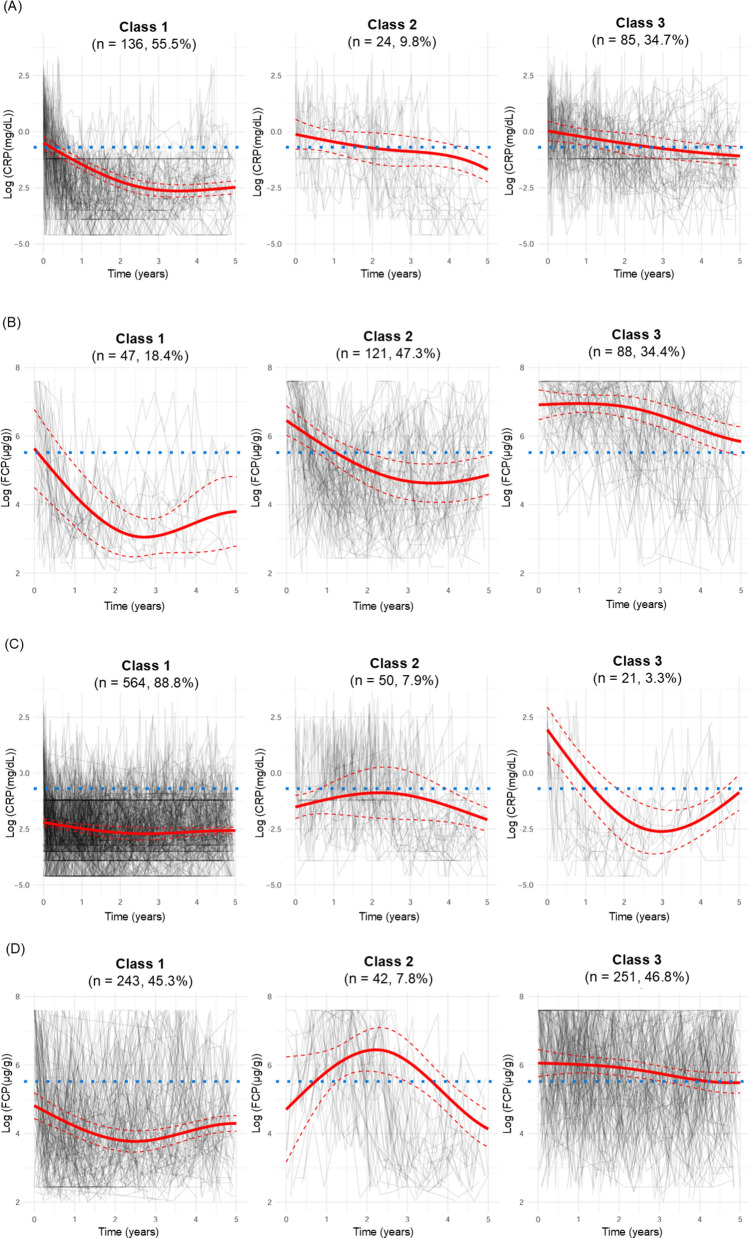
C-reactive protein (CRP) and fecal calprotectin (FCP) trajectories from inflammatory bowel disease diagnosis in patients with Crohn’s disease (CD) or ulcerative colitis (UC). Log-transformed (**A**) CRP and (**B**) FCP trajectories for patients with CD. Log-transformed (**C**) CRP and (**D**) FCP trajectories for patients with UC. The solid red line represents the predicted mean trajectory for each class, and the red dotted lines indicate the 95% confidence intervals. The gray lines indicate the trajectories of each patient. The blue dotted line represents an FCP value of log (250 μg/g) or CRP value of log (0.5 mg/dL)

The three classes differed in terms of longitudinal trends in CRP or FCP levels (Fig. 1). In general, class 1 patients exhibited relatively low CRP or FCP levels throughout the follow-up period, indicating that these patients quickly and easily achieved biochemical remission. Patients in class 3, however, had relatively high CRP or FCP levels, suggesting that these patients struggled to achieve biochemical improvement. The CRP or FCP values for class 2 were intermediate, higher than those of class 1 but lower than those of class 3.

For patients with CD, the CRP trajectories were categorized into three classes: class 1 showed rapidly decreasing and consistently low trajectories, class 2 displayed gradually decreasing trajectories, and class 3 exhibited slowly decreasing trajectories, taking several years to reach the target CRP level of 0.5 mg/dL. The FCP trajectories in patients with CD were categorized as follows: class 1 showed rapidly decreasing and substantially low trajectories, class 2 displayed gradually decreasing trajectories, and class 3 exhibited high initial trajectories that failed to reach the target of 250 μg/g even after 5 years. In patients with UC, class 1 CRP trajectories consisted of stable low trajectories, class 2 exhibited moderate-stable trajectories, and class 3 initially displayed high and U-shaped trajectories. The FCP trajectories in patients with UC were classified as follows: class 1 showed stable low trajectories, class 2 exhibited inverted U-shape trajectories, and class 3 had relatively high levels that were challenging to bring below the target threshold over 5 years.

### Univariate and multinomial logistic regression analyses of classes according to biochemical markers in patients with CD

In the analysis of longitudinal CRP trends in CD, classes 1, 2, and 3 comprised 136 (55.5%), 24 (9.8%), and 85 (34.7%) patients, respectively (Table 1). In the univariate analyses comparing the early initiation of IM or AT, early ER visits, and early hospitalization among the three classes, the *p*-values were < 0.200. Early IM treatment was the only significant predictor distinguishing class 1 from classes 2 and 3, and patients with early IM treatment were less likely to be classified as class 2 or 3 than class 1 (class 2 vs. 1, odds ratio [OR] = 0.344, 95% confidence interval [CI] = 0.127–0.931; class 3 vs. 1, OR = 0.472, 95% CI = 0.229–0.971; *p*-value = 0.043).

**Table 1:** Clinical characteristics, univariate analysis, and multinomial logistic regression analysis based on longitudinal trajectories of C-reactive protein levels in patients with Crohn’s disease

	Total patients	Univariate analyses				Multinomial logistic regression analysis		
		Class 1	Class 2	Class 3	*p*-value^a^	OR (95% CI)		
						Class 2^b^	Class 3^b^	*p*-value^a^
Number	245	136 (55.5)	24 (9.8)	85 (34.7)				
Female	52 (21.2)	27 (19.9)	5 (20.8)	20 (23.5)	0.808			
Age at diagnosis, years	25.1 (9.7)	24.7 (9.3)	25.0 (11.3)	25.9 (9.8)	0.693			
Current smoker	37 (15.1)	18 (13.2)	3 (12.5)	16 (18.8)	0.493			
History of appendectomy	21 (8.6)	10 (7.4)	2 (8.3)	9 (10.6)	0.704			
*CCI*								
0	229 (93.5)	130 (95.6)	23 (95.8)	76 (89.4)	0.353			
1	15 (6.1)	6 (4.4)	1 (4.2)	8 (9.4)				
≥ 2	1 (0.4)	0 (0.0)	0 (0.0)	1 (1.2)				
Positive ANCA titer	3 (2.3)	1 (1.3)	0 (0.0)	2 (5.1)	0.352			
Elevation of ASCA IgG or IgA	147 (60.2)	83 (61.0)	15 (62.5)	49 (58.3)	0.898			
*Disease location*								
Ileal	61 (24.9)	38 (27.9)	6 (25.0)	17 (20.0)	0.659			
Colonic	16 (6.5)	10 (7.4)	1 (4.2)	5 (5.9)				
Ileocolonic	168 (68.6)	88 (64.7)	17 (70.8)	63 (74.1)				
UGI involvement	31 (12.7)	18 (13.2)	4 (16.7)	9 (10.6)	0.698			
*Disease behavior*								
Non-stricturing, non-penetrating	156 (63.7)	87 (64.0)	16 (66.7)	53 (62.4)	0.550			
Stricturing	32 (13.1)	15 (11.0)	5 (20.8)	12 (14.1)				
Penetrating	57 (23.3)	34 (25.0)	3 (12.5)	20 (23.5)				
Perianal disease	122 (49.8)	65 (47.8)	12 (50.0)	45 (52.9)	0.758			
Early CS	158 (64.5)	87 (64.0)	14 (58.3)	57 (67.1)	0.720			
Early IM	198 (80.8)	118 (86.8)	16 (66.7)	64 (75.3)	**0.019**	0.344 (0.127–0.931)	0.472 (0.229–0.971)	**0.039**
Early AT	100 (40.8)	44 (32.4)	5 (20.8)	18 (21.2)	**0.145**	0.742 (0.251–2.198)	0.495 (0.249–0.984)	0.120
Early intestinal resection	11 (4.5)	6 (4.4)	0 (0.0)	5 (5.9)	0.469			
Early ER visit	40 (16.3)	20 (14.7)	1 (4.2)	19 (22.4)	**0.077**	0.406 (0.040–4.158)	2.102 (0.791–5.585)	0.200
Early hospitalization	49 (20.0)	26 (19.1)	2 (8.2)	21 (24.7)	**0.194**	0.691 (0.125–3.817)	1.156 (0.474–2.817)	0.800

CCI, Charlson comorbidity index; ANCA, anti-neutrophil cytoplasmic antibody; ASCA, anti-*Saccharomyces cerevisiae* antibody; CS, corticosteroid, IM, immunomodulator; AT, advanced therapy; ER, emergency room

Values are presented as the mean (standard deviation) or number (%)

^a^ *p*-value is indicated in bold when < 0.200 in univariate analyses and when < 0.050 in multinomial logistic regression analysis

^b^ All groups were compared with class 1

In the analysis of longitudinal FCP trends in CD, classes 1, 2, and 3 included 47 (18.4%), 121 (47.3%), and 88 (34.4%) patients, respectively (Table 2). In the univariate analyses comparing the history of appendectomy, upper gastrointestinal involvement, perianal disease, early AT, early ER visits, and early hospitalization among the three classes, the *p*-values were < 0.200. In the multinomial logistic regression, early initiation of AT was the only significant predictor distinguishing class 1 from classes 2 and 3 (class 2 vs. 1, OR = 0.383, 95% CI = 0.170–0.863; class 3 vs. 1, OR = 0.220, 95% CI = 0.089–0.545; *p*-value = 0.004). Patients with a history of early ER visits had higher odds of belonging to class 3 than to class 1 (OR = 6.598, 95% CI = 1.116–39.00), whereas early ER visits did not change the likelihood of belonging to class 1 or 2.

**Table 2:** Clinical characteristics, univariate analysis, and multinomial logistic regression analysis based on different longitudinal trajectories of fecal calprotectin levels in patients with Crohn’s disease

	Total patients	Univariate analyses				Multinomial logistic regression analysis		
		Class 1	Class 2	Class 3	*p*-value^a^	OR (95% CI)		*p*-value^a^
						Class 2^b^	Class 3^b^	
Number	256	47 (18.4)	121 (47.3)	88 (34.4)				
Female	53 (20.7)	10 (21.3)	23 (19.0)	20 (22.7)	0.802			
Age at diagnosis, years	25.2 (15.4)	24.0 (8.0)	24.7 (10.1)	26.5 (10.0)	0.265			
Current smoker	37 (14.5)	5 (10.6)	15 (12.4)	17 (19.3)	0.266			
History of appendectomy	22 (8.6)	1 (2.1)	11 (9.1)	10 (11.4)	**0.183**	4.068 (0.479–34.53)	5.806 (0.676–49.88)	0.200
*CCI*								
0	240 (93.8)	45 (95.7)	115 (95.0)	80 (90.9)	0.548			
1	15 (5.9)	2 (4.3)	6 (5.0)	7 (8.0)				
≥ 2	1 (0.4)	0 (0.0)	0 (0.0)	1 (1.1)				
Positive ANCA titer	3 (2.3)	0 (0.0)	2 (1.7)	1 (1.1)	0.573			
Elevation of ASCA IgG or IgA	154 (60.4)	24 (51.1)	76 (63.3)	54 (61.4)	0.336	1.846 (0.895–3.807)	1.782 (0.828–3.833)	0.200
*Disease location*								
Ileal	66 (25.8)	11 (23.4)	35 (28.9)	20 (22.7)	0.258			
Colonic	16 (6.3)	6 (12.8)	5 (4.1)	5 (5.7)				
Ileocolonic	174 (68.0)	30 (63.8)	81 (66.9)	63 (71.6)				
UGI involvement	35 (13.7)	4 (8.5)	14 (11.6)	17 (19.3)	**0.143**	1.474 (0.442–4.917)	2.865 (0.865–9.490)	0.110
*Disease behavior*								
Non-stricturing, non-penetrating	163 (63.7)	33 (70.2)	77 (63.6)	53 (60.2)	0.551			
Stricturing	35 (13.7)	5 (10.6)	14 (11.6)	16 (18.2)				
Penetrating	58 (22.7)	9 (19.1)	30 (24.8)	19 (21.6)				
Perianal disease	128 (50.0)	26 (55.3)	53 (43.8)	49 (55.7)	**0.171**	0.724 (0.350–1.496)	1.245 (0.577–2.688)	0.200
Early CS	161 (62.9)	28 (59.6)	79 (65.3)	54 (61.4)	0.738			
Early IM	209 (81.4)	42 (89.4)	98 (81.0)	69 (78.4)	0.284			
Early AT	68 (26.6)	18 (38.3)	33 (27.3)	17 (19.3)	**0.057**	0.383 (0.170–0.863)	0.220 (0.089–0.545)	**0.004**
Early intestinal resection	11 (4.3)	1 (2.1)	8 (6.6)	2 (2.3)	0.224			
Early ER visit	42 (16.4)	2 (4.3)	23 (19.0)	17 (19.3)	**0.045**	4.247 (0.773–23.34)	6.598 (1.116–39.00)	0.071
Early hospitalization	20 (19.5)	4 (8.5)	29 (24.0)	17 (19.3)	**0.076**	2.282 (0.628–8.297)	1.465 (0.363–5.913)	0.300

CCI, Charlson comorbidity index; ANCA, anti-neutrophil cytoplasmic antibody; ASCA, anti-*Saccharomyces cerevisiae* antibody; CS, corticosteroid, IM, immunomodulator; AT, advanced therapy; ER, emergency room

Values are presented as the mean (standard deviation) or number (%)

^a^ *p*-value is indicated in bold when < 0.200 in univariate analyses and when < 0.050 in multinomial logistic regression analysis

^b^ All groups were compared with class 1

### Univariate and multinomial logistic regression analyses of biochemical marker-based classes in patients with UC

Most patients were classified into class 1 (*n* = 564, 88.8%), with 7.9% (*n* = 50) and 3.3% (*n* = 21) classified into classes 2 and 3, respectively (Table 3). Significant differences (*p*-value < 0.200) among the classes were observed regarding age at diagnosis; positive ANCA titer; disease extent; early treatment with CS, IM, and AT; early ER visits; and early hospitalization. Unlike the analyses for CD, in multinomial logistic regression, no significant predictors of class membership were identified based on the CRP latent classes in patients with UC.

**Table 3:** Clinical characteristics, univariate analysis, and multinomial logistic regression analysis based on different longitudinal trajectories of C-reactive protein levels in patients with ulcerative colitis

	Total patients	Univariate analyses				Multinomial logistic regression analysis		
		Class 1	Class 2	Class 3	*p*-value^a^	OR (95% CI)		*p*-value^a^
						Class 2^b^	Class 3^b^	
Number	635	564 (88.8)	50 (7.9)	21 (3.3)				
Female	234 (36.9)	212 (37.6)	16 (32.0)	6 (28.6)	0.534			
Age at diagnosis, years	38.3 (14.8)	37.8 (14.0)	43.2 (19.0)	39.2 (15.3)	**0.037**	1.026 (0.998–1.054)	1.027 (0.989–1.067)	0.094
Current smoker	101 (15.9)	92 (16.3)	5 (10.0)	4 (19.0)	0.466			
History of appendectomy	17 (2.7)	14 (2.5)	3 (6.0)	0 (0.0)	0.249			
*CCI*								
0	527 (83.0)	467 (82.8)	41 (82.0)	19 (90.5)	0.896			
1	74 (11.7)	67 (11.9)	6 (12.0)	1 (4.8)				
≥ 2	34 (5.4)	30 (5.3)	3 (6.0)	1 (4.8)				
Positive ANCA titer	57 (14.0)	44 (12.2)	10 (33.3)	3 (18.8)	**0.005**	2.918 (1.185–7.187)	0.858 (0.204–3.604)	0.073
Elevation of ASCA IgG or IgA	100 (17.9)	86 (17.2)	10 (25.0)	4 (22.2)	0.414			
*Disease extent*								
Proctitis	246 (38.7)	226 (40.1)	15 (30.0)	5 (23.8)	**0.054**			0.400
Left-sided colitis	179 (28.2)	162 (28.7)	13 (26.0)	4 (19.0)		1.334 (0.472–3.770)	0.517 (0.075–3.557)	
Extensive colitis	210 (33.1)	176 (31.2)	22 (44.0)	12 (57.1)		1.425 (0.482–4.216)	1.958 (0.427–8.975)	
Early CS	199 (31.3)	168 (29.8)	19 (38.0)	12 (57.1)	**0.017**	0.897 (0.330–2.441)	2.111 (0.536–8.315)	0.500
Early IM	149 (23.5)	123 (21.8)	14 (28.0)	12 (57.1)	**0.001**	0.830 (0.302–2.280)	3.610 (1.120–11.64)	0.085
Early AT	11 (1.7)	3 (0.5)	6 (12.0)	2 (9.5)	** < 0.001**	9.759 (1.024–92.99)	4.865 (0.488–48.46)	0.110
Early ER visit	42 (6.6)	31 (5.5)	7 (14.0)	4 (19.0)	**0.004**	0.991 (0.150–6.563)	1.537 (0.175–13.50)	> 0.999
Early hospitalization	36 (5.7)	24 (4.3)	8 (16.0)	4 (19.0)	** < 0.001**	4.099 (0.678–24.78)	2.634 (0.276–25.18)	0.300

CCI, Charlson comorbidity index; ANCA, anti-neutrophil cytoplasmic antibody; ASCA, anti-*Saccharomyces cerevisiae* antibody; CS, corticosteroid, IM, immunomodulator; AT, advanced therapy; ER, emergency room

Values are presented as the mean (standard deviation) or number (%)

^a^ *p*-value is indicated in bold when < 0.200 in univariate analyses and when < 0.050 in multinomial logistic regression analysis

^b^ All groups were compared with class 1

In the analysis of longitudinal FCP trends in UC, most patients were classified into class 1 (*n* = 243, 45.3%) or class 3 (*n* = 251, 46.8%), with a small number classified into class 2 (*n* = 42, 7.8%) (Table 4). Significant associations (*p*-value < 0.200) with longitudinal FCP trends were identified for age at diagnosis, smoking status, history of appendectomy, elevated ASCA IgG or IgA levels, and early CS and IM treatment. Younger age at diagnosis (class 2 vs. 1, OR = 0.969, 95% CI = 0.944–0.995; class 3 vs. 1, OR = 0.980, 95% CI = 0.966–0.994; *p*-value = 0.004) and early IM treatment (class 2 vs. 1, OR = 2.928, 95% CI = 1.289–6.651; class 3 vs. 1, OR = 2.698, 95% CI = 1.657–4.394; *p*-value < 0.001) were associated with higher odds of belonging to class 2 or 3. In contrast, current smoking increased the likelihood of being in class 1 (class 2 vs. 1, OR = 0.194, 95% CI = 0.044–0.846; class 3 vs. 1, OR = 0.559, 95% CI = 0.330–0.945; *p*-value = 0.008).

**Table 4:** Clinical characteristics, univariate analysis, and multinomial logistic regression analysis based on different longitudinal trajectories of fecal calprotectin levels in patients with ulcerative colitis

	Total patients	Univariate analyses				Multinomial logistic regression analysis		
		Class 1	Class 2	Class 3	*p*-value^a^	OR (95% CI)		*p*-value^a^
						Class 2^b^	Class 3^b^	
Number	536	243 (45.3)	42 (7.8)	251 (46.8)				
Female	198 (36.9)	94 (38.7)	19 (45.2)	85 (33.9)	0.276			
Age at diagnosis, years	38.7 (14.2)	41.0 (12.8)	35.7 (16.2)	37.0 (15.3)	**0.003**	0.969 (0.944–0.995)	0.980 (0.966–0.994)	**0.004**
Current smoker	84 (15.7)	48 (19.8)	2 (4.8)	34 (13.5)	**0.021**	0.194 (0.044–0.846)	0.559 (0.330–0.945)	**0.008**
History of appendectomy^c^	15 (2.8)	10 (4.1)	0 (0)	5 (2.0)	**0.187**		0.420 (0.133–1.325)	0.091
*CCI*								
0	443 (82.6)	202 (83.1)	37 (88.1)	204 (81.3)	0.837			
1	64 (11.9)	29 (11.9)	3 (7.1)	32 (12.7)				
≥ 2	29 (5.4)	12 (4.9)	2 (4.8)	15 (6.0)				
Positive ANCA titer	51 (9.5)	19 (7.8)	4 (9.5)	28 (11.2)	0.506			
Elevation of ASCA IgG or IgA	86 (16.0)	31 (14.0)	8 (20.5)	47 (20.8)	**0.154**	1.220 (0.501–2.972)	1.384 (0.824–2.325)	0.500
*Disease extent*								
Proctitis	210 (39.2)	102 (42.0)	19 (45.2)	89 (35.5)	0.277			
Left-sided colitis	151 (28.2)	69 (28.4)	13 (31.0)	69 (27.5)				
Extensive colitis	175 (32.6)	72 (29.6)	10 (23.8)	93 (37.1)				
Early CS	165 (30.8)	61 (25.1)	15 (35.7)	89 (35.5)	**0.034**	1.032 (0.470–2.267)	1.030 (0.662–1.604)	> 0.999
Early IM	36 (14.8)	36 (14.8)	15 (35.7)	80 (31.9)	** < 0.001**	2.928 (1.289–6.651)	2.698 (1.657–4.394)	** < 0.001**
Early AT	11 (2.1)	3 (1.2)	1 (2.4)	7 (2.8)	0.470			
Early ER visit	35 (6.5)	14 (5.8)	3 (7.1)	18 (7.2)	0.806			
Early hospitalization	29 (5.4)	10 (4.1)	2 (4.8)	17 (6.8)	0.419			

CCI, Charlson comorbidity index; ANCA, anti-neutrophil cytoplasmic antibody; ASCA, anti-*Saccharomyces cerevisiae* antibody; CS, corticosteroid, IM, immunomodulator; AT, advanced therapy; ER, emergency room

Values are presented as the mean (standard deviation) or number (%)

^a^ *p*-value is indicated in bold when < 0.200 in univariate analyses and when < 0.050 in multinomial logistic regression analysis

^b^ All groups were compared with class 1

^c^ Classes 1 and 2 could not be compared because no patients in class 2 had a history of appendectomy

### Sensitivity analysis

Sensitivity analysis was conducted on patients with a likelihood of > 70% for inclusion in specific latent classes. The analysis of demographic and clinical factors, restricted to these patients, produced results consistent with those observed for the entire cohort (Supplementary Tables 2–5). Early IM initiation was identified as the only significant predictor of belonging to class 1 in the CRP LCA in the multinomial logistic regression analysis of patients with CD, with a likelihood of > 70% for classification into each class. These results are consistent with those of the analysis that included all patients with CD. Early AT initiation was a significant predictor of belonging to class 1 in the FCP LCA in the multinomial logistic regression for patients with CD, with a likelihood of > 70% for classification into each class. These results are consistent with the results of the analysis that included all patients with CD. Patients with a history of early ER visits were more likely to be included in class 2 or 3 than in class 1. However, given that no patients in class 1 had early ER visits, the reliability of the analysis was limited.

The results of the multinomial logistic regression analysis focusing only on patients with UC with a likelihood of > 70% belonging to each class were consistent with those obtained from the analysis of all patients with UC. No significant predictors of specific class membership were observed in the CRP LCA of patients with UC, with a likelihood of > 70% for each class. Conversely, younger age at diagnosis was significantly associated with higher odds of belonging to class 2 or 3 in the FCP LCA of patients with UC, with a likelihood of > 70% for classification into each class. The current smoking was significantly associated with higher odds of belonging to class 1 than to class 3. Early IM treatment significantly increased the likelihood of belonging to class 2 or 3.

### Clinical outcomes according to biochemical marker-based classes in patients with IBD

IBD-related ER visits, hospitalizations, and intestinal resections over a 5-year follow-up period based on CRP and FCP trajectories in patients with CD and UC were analyzed (Supplementary Table 6). In the analyses of CRP trajectories for CD, ER visits and hospitalizations were significantly different across classes (*p*-value = 0.039 and *p*-value = 0.005, respectively), with Class 3 having the highest proportions (38.8% and 43.5%, respectively). Similarly, in the FCP trajectory analysis for CD, ER visits and hospitalizations also showed significant differences across classes (*p*-value = 0.001 and *p*-value = 0.002, respectively), with Class 3 having the highest proportions (37.5% and 39.8%, respectively). In UC, CRP and FCP trajectory analyses showed significant differences in ER visits (*p*-value < 0.001 and *p*-value = 0.009, respectively) and hospitalizations (*p*-value < 0.001 and *p*-value = 0.002, respectively). However, unlike in CD, Class 2, not Class 3, had the highest proportions for ER visits (50.0% and 31.0%, respectively) and hospitalizations (52.0% and 23.8%, respectively). No significant differences in the proportions of intestinal resections were observed across the groups.

## Discussion

Our study employed the LCMM to classify the long-term trends in biochemical markers in patients with IBD. Three distinct subclasses of CRP and FCP levels were identified for CD and UC, revealing different clinical characteristics across different CRP and FCP trajectories. Moreover, higher CRP and FCP trajectory classes were associated with worse long-term outcomes, including IBD-related ER visits and hospitalizations.

Although both CRP and FCP serve as valuable biomarkers in IBD, they have distinct characteristics. CRP has relatively low sensitivity, resulting in cases where patients with active inflammation exhibit low CRP levels [27]. In contrast, FCP demonstrates significantly higher sensitivity and is more reliable in detecting endoscopically active inflammation. Therefore, CRP and FCP trajectories may exhibit different patterns even within the same class.

In the CRP trajectories of patients with CD, classes 2 and 3 did not achieve reduced CRP levels to the target levels recommended by STRIDE II as early as class 1. However, they eventually reached the target level over several years. In contrast, the proportion of patients who achieved biochemical remission shortly after diagnosis was significantly lower in the FCP trajectory than in the CRP trajectory (class 1: 18.4% vs. 55.5%). Moreover, approximately one-third of the patients (class 3) struggled to reach the target FCP level even after 5 years. Despite progress in advanced therapies, long-term reductions in FCP levels in patients with CD remain challenging.

Early IM treatment for CD has been shown to improve clinical outcomes compared with conventional therapy [28–30]. Compared with delayed treatment, early initiation of biologics in patients with CD is associated with a lower risk of CD-related surgery [30, 31]. Additionally, the top-down strategy is superior to the step-up strategy in terms of the prognosis of patients with CD [31, 32]. In this study, patients with CD who received early IM treatment were likely to have low CRP trajectories, and those who received early AT were likely to have low FCP trajectories. Early initiation of IM and AT in CD was associated with long-term biochemical remission, indicating that they can improve the long-term disease course in patients with CD.

CRP trajectories can be reduced by early IM treatment alone in patients with CD. However, decreasing the FCP trajectories requires early AT and cannot be achieved by early IM treatment alone. This result highlights the following: in the CRP analysis, the proportion of patients in class 1 was higher than that in class 2 or 3, whereas in the FCP analysis, the proportion of patients in class 2 or 3 was higher than that in class 1. This result indicates that although STRIDE-II recommends CRP and FCP as short- to medium-term treatment goals [1], it is more challenging to reduce FCP levels below the target than to normalize CRP levels. Additionally, the findings of this study suggest that early IM treatment alone is insufficient to achieve a favorable prognosis in patients with severe CD and that early AT is required. A study involving pediatric patients with CD showed that early anti-TNF treatment was superior to early IM treatment in achieving clinical remission and promoting growth [33], supporting the results of this study.

In the CRP trajectory of patients with UC, only 3.3% of patients (class 3) had elevated CRP levels at diagnosis. Most patients had low CRP levels at diagnosis, and their CRP levels did not increase remarkably above the target level during the 5-year follow-up. Unlike patients with CD, those with UC did not have any variables that significantly differed along the CRP trajectories. CRP responses were more pronounced in patients with CD than in those with UC [34], likely due to the generally lower CRP levels in patients with UC than in those with CD [35]. Therefore, CRP may be a more reliable biochemical marker for assessing the long-term prognosis of CD than that of UC. In patients with mild UC, CRP levels are likely to be normal, regardless of disease activity, making FCP a more valuable and reliable biochemical marker than CRP for identifying and pursuing treatment targets.

Early IM treatment significantly increased the risk of a high FCP trajectory in patients with UC. Considering that this was an observational study, patients with severe disease activity may require IM treatment soon after diagnosis. In previous studies, early IM treatment in patients with UC did not reduce the risk of colectomy compared with delayed IM treatment [36–38], similar to the current study. However, early AT does not significantly affect FCP trajectories in patients with UC. Although the early initiation of biologics in patients with CD contributes to achieving remission and reducing the risk of bowel resection, whether early initiation of biologics is beneficial for patients with UC remains unclear [39, 40]. Further research is required to determine whether this result is owing to the small number of patients who received early AT in this study or whether early AT cannot improve long-term outcomes of UC.

This study had several strengths. First, CD and UC were examined using the same methodological approaches, allowing for a comprehensive understanding of disease dynamics and long-term outcomes across the spectrum of IBD. Additionally, CRP and FCP levels, which are considered intermediate goals for IBD treatment, were analyzed. Second, the LCMM was used to classify patients with CD and UC into distinct classes based on longitudinal trends in CRP and FCP levels. This method provides a more objective and detailed analysis of disease progression than previous studies, which relied on subjective patient-reported outcomes. Third, this study highlighted the effect of early aggressive medical treatment on long-term biochemical outcomes in patients with CD. Early aggressive medical treatment in patients with CD is beneficial [4, 41, 42]; however, previous studies evaluated the effectiveness of early aggressive medical treatment based on the patient’s symptoms, complications, and endoscopic mucosal healing but did not assess it using objective biochemical markers. In contrast, our study revealed that early aggressive medical treatment contributed to biochemical improvement. Additionally, this study evaluated the outcomes over a more extended period of 5 years, whereas previous studies have assessed the outcomes of early aggressive medical treatment over a maximum period of 2 years [42].

However, this study had some limitations. First, the intervals of the CRP and FCP measurements were not consistent across all patients. However, when examining testing intervals by class, higher classes did not necessarily correspond to more frequent testing. Additionally, we applied LCMMs to account for the variability in the intervals of CRP and FCP testing among patients. By using restricted cubic splines to model testing intervals over time, we obtained a smoothed representation that accommodated this variability. This approach effectively incorporates individual variations into the model’s optimized curve. Moreover, the model fit tests confirmed that this method accurately captured the inter-patient variability and ensured robust model performance over time. Second, although the LCMMs were used to classify patients based on biochemical marker trajectories, the interpretation of these trajectories relied on statistical assumptions and model selection criteria. To mitigate this issue, a sensitivity analysis was performed that only focused on patients with > 70% probability of belonging to specific classes. The findings of this subgroup analysis closely resembled those obtained from studies involving all patients. Third, as this is a retrospective and observational study, it cannot establish causality but can identify associations. However, the finding that early AT increased the probability of belonging to class 1 in the FCP trajectory analysis for CD is consistent with the results of a prospective study demonstrating that early AT in CD can improve 1-year outcomes [41].

In conclusion, three distinct classes were identified for each biochemical marker (CRP or FCP) in CD and UC, reflecting varying disease courses. In patients with CD, early aggressive medical treatment was associated with favorable long-term biochemical marker trajectories. However, early IM treatment alone is insufficient, and early AT is required to achieve deeper biochemical remission. Furthermore, whether early aggressive medical treatment improves the disease course of UC based on biochemical activity is unclear. Therefore, timely and targeted interventions based on a thorough understanding of biochemical markers could markedly improve disease courses and patient outcomes.

## Supplementary Information

Below is the link to the electronic supplementary material.

Supplementary file1 (DOCX 63 KB) Supplementary tables

Supplementary file2 (TIF 244 KB) Supplementary Fig. 1. Standards for Reporting of Diagnostic Accuracy (STARD) diagram for the study. The flow diagram illustrates the recruitment and classification of study participants according to the STARD guideline

Supplementary file3 (TIF 2285 KB) Supplementary Fig. 2. Alluvial plots show how the distribution of class membership among patients changed with varying numbers of C-reactive protein or fecal calprotectin trajectory classes. (A) Alluvial plot of C-reactive protein (CRP) trajectory models for patients with Crohn’s disease (CD). (B) Alluvial plot of fecal calprotectin (FCP) trajectory models for patients with CD. (C) Alluvial plot of CRP trajectory models for patients with ulcerative colitis (UC). (D) Alluvial plot of FCP trajectory models for patients with UC

Supplementary file4 (TIF 91 KB) Supplementary Fig. 3. Comparison of testing intervals across classes using box plots. The bottom and top edges of the box represent the first (Q1) and third quartiles (Q3), indicating the 25 th and 75 th percentiles of the data, respectively. The line inside the box represents the median (Q2), which corresponds to the 50 th percentile. The whisker extends from the bottom or top of the box to the smallest or largest data point, which is 1.5 times the interquartile range from Q1 or Q3. Individual points outside the whiskers are considered outliers. Although outliers are present, the position and size of the boxes do not differ noticeably across classes. (A) A box plot of C-reactive protein (CRP) testing intervals in patients with Crohn’s disease (CD). (B) A box plot of fecal calprotectin (FCP) testing intervals in patients with CD. (C) A box plot of CRP testing intervals in patients with ulcerative colitis (UC). (D) A box plot of FCP testing intervals in patients with UC
