# Supplementary material for: Impact of early aggressive treatment on long-term biochemical marker patterns in inflammatory bowel disease
Source: J Gastroenterol. 2025 May 2;60(7):854–65. doi: 10.1007/s00535-025-02244-w (PMC12176982; doi:10.1007/s00535-025-02244-w)
Supplement: Supplementary file 1 — Supplementary file1 (DOCX 63 KB) Supplementary tables [file 535_2025_2244_MOESM1_ESM.docx]

**Supplementary Table 1.** Model fit statistics for latent class models using C-reactive protein or fecal calprotectin data in patients with different numbers of latent subclasses

| CRP models in patients with CD | | | |
| --- | --- | --- | --- |
| Class | **Maximum log-likelihood** | **AIC** | **BIC** |
| 1 | -12962.16 | 25966.32 | 26039.85^a^ |
| 2 | -12954.04 | 25962.08 | 26056.62 |
| 3 | -12948.66 | 25963.33 | 26078.87 |
| 4 | -12939.36 | 25956.72 | 26093.27 |
| 5 | -12943.18 | 25976.37 | 26133.92 |
| 6 | -12890.74^a^ | 25883.47^a^ | 26062.04 |
| FCP models in patients with CD | | | |
| Class | **Maximum log-likelihood** | **AIC** | **BIC** |
| 1 | -6836.28 | 13714.57 | 13789.02 |
| 2 | -6828.24 | 13710.48 | 13806.2 |
| 3 | -6822.96 | 13711.92 | 13828.91 |
| 4 | -6814.63 | 13707.26 | 13845.52 |
| 5 | -6807.4 | 13704.8 | 13864.33 |
| 6 | -6800.31 | 13702.61 | 13883.42 |
| CRP models in patients with UC | | | |
| Class | **Maximum log-likelihood** | **AIC** | **BIC** |
| 1 | -21667.81 | 43377.61 | 43471.14 |
| 2 | -21627.97 | 43309.93 | 43430.18 |
| 3 | -21615.24 | 43296.48 | 43443.45 |
| 4 | -21589.07 | 43256.14 | 43429.83 |
| 5 | -21582.03 | 43254.07 | 43454.48 |
| 6 | -21553.88 | 43209.77 | 43436.9 |
| FCP models in patients with UC | | | |
| Class | **Maximum log-likelihood** | **AIC** | **BIC** |
| 1 | -11028.32 | 22098.64 | 22188.61 |
| 2 | -11016.33 | 22086.67 | 22202.34 |
| 3 | -11009.04 | 22084.08 | 22225.45 |
| 4 | -11004.37 | 22086.75 | 22253.83 |
| 5 | -10997.39 | 22084.77 | 22277.56 |
| 6 | -10990.62 | 22083.24 | 22301.73 |

AIC, Alkaike information criterion; BID, Bayesian information criterion; CRP, C-reactive protein; FCP, fecal calprotectin; CD, Crohn’s disease; UC, ulcerative colitis

**Supplementary Table 2.** Clinical characteristics, univariate analysis, and multinomial logistic regression analysis based on the longitudinal trajectories of C-reactive protein in patients with Crohn’s disease with a probability of trajectory class membership > 70%

|  | | Total patients | Univariate analyses | | | | Multinomial logistic regression analysis | | |
| --- | --- | --- | --- | --- | --- | --- | --- | --- | --- |
|  |  |  | Class 1 | Class 2 | Class 3 | *p*-value^a^ | OR (95% CI) | | |
|  |  |  |  |  |  |  | Class 2^b^ | Class 3^b^ | *p*-value^a^ |
| Number | | 179 | 114 (63.7) | 19 (10.6) | 46 (24.7) |  |  |  |  |
| Female | | 39 (21.8) | 23 (20.2) | 5 (26.3) | 11 (23.9) | 0.769 |  |  |  |
| Age at diagnosis, years | | 24.9 (9.7) | 24.6 (9.2) | 25.9 (12.3) | 25.4 (10.0) | 0.832 |  |  |  |
| Current smoker | | 26 (14.5) | 14 (12.3) | 3 (15.8) | 9 (19.6) | 0.490 |  |  |  |
| History of appendectomy | | 16 (8.9) | 7 (6.1) | 2 (10.5) | 7 (15.2) | **0.184** | 2.171  (0.377–12.49) | 1.782  (0.531–5.979) | 0.500 |
| CCI | 0 | 166 (92.7) | 108 (94.7) | 18 (94.7) | 40 (87.0) | 0.318 |  |  |  |
|  | 1 | 12 (6.7) | 6 (5.3) | 1 (5.3) | 5 (10.9) |  |  |  |  |
|  | ≥ 2 | 1 (0.6) | 0 (0.0) | 0 (0.0) | 1 (2.2) |  |  |  |  |
| Positive ANCA titer | | 2 (2.2) | 1 (1.6) | 0 (0.0) | 1 (5.3) | 0.562 |  |  |  |
| Elevation of ASCA IgG or IgA | | 106 (59.8) | 70 (61.4) | 12 (63.2) | 24 (54.3) | 0.677 |  |  |  |
| Disease location | Ileal | 43 (24.0) | 29 (25.4) | 5 (26.3) | 9 (19.6) | 0.805 |  |  |  |
|  | Colonic | 12 (6.7) | 9 (7.9) | 1 (5.3) | 2 (4.3) |  |  |  |  |
|  | Ileocolonic | 124 (69.3) | 76 (66.7) | 13 (68.4) | 35 (76.1) |  |  |  |  |
| UGI involvement | | 23 (12.8) | 16 (14.0) | 1 (5.3) | 6 (13.0) | 0.571 |  |  |  |
| Disease behavior | Nonstricturing, nonpenetrating | 115 (64.2) | 75 (65.8) | 12 (63.2) | 28 (60.9) | 0.500 |  |  |  |
|  | Stricturing | 20 (11.2) | 12 (10.5) | 4 (21.1) | 4 (8.7) |  |  |  |  |
|  | Penetrating | 44 (24.6) | 27 (23.7) | 3 (15.8) | 14 (30.4) |  |  |  |  |
| Perianal disease | | 86 (48.0) | 53 (46.5) | 9 (47.4) | 24 (52.2) | 0.807 |  |  |  |
| Early CS | | 116 (64.8) | 71 (62.3) | 11 (57.9) | 34 (73.9) | 0.303 |  |  |  |
| Early IM | | 142 (79.3) | 97 (85.1) | 12 (63.2) | 33 (71.7) | **0.031** | 0.350  (0.119–1.025) | 0.340  (0.142–0.811) | **0.022** |
| Early AT | | 52(29.1) | 37 (32.5) | 4 (21.1) | 11 (23.9) | 0.402 |  |  |  |
| Early intestinal resection^c^ | | 9 (5.0) | 4 (3.5) | 0 (0.0) | 5 (10.9) | **0.089** |  | 2.426  (0.547–10.76) | 0.300 |
| Early ER visit | | 30 (16.8) | 15 (13.2) | 1 (5.3) | 14 (30.4) | **0.011** | 0.774  (0.068–8.771) | 1.979  (0.623–6.287) | 0.500 |
| Early hospitalization | | 41 (22.9) | 23 (20.2) | 1 (5.3) | 17 (37.0) | **0.011** | 0.294  (0.029–2.933) | 1.601  (0.560–4.576) | 0.300 |

| CCI, Charlson Comorbidity Index; ANCA, anti-neutrophil cytoplasmic antibody; ASCA, anti-*Saccharomyces cerevisiae* antibody; CS, corticosteroid, IM, immunomodulator; AT, advanced therapy; ER, emergency room  Values are presented as the mean (standard deviation) or number (%).  ^a^ *p*-value is indicated in bold when < 0.200 in univariate analyses and when < 0.050 in multinomial logistic regression analysis.  ^b^ All groups were compared with class 1.  ^c^ Classes 1 and 2 could not be compared because no patients in class 2 had early intestinal resection. |
| --- |

**Supplementary Table 3.** Clinical characteristics, univariate analyses, and multinomial logistic regression analysis based on longitudinal trajectories of fecal calprotectin in patients with Crohn’s disease with a probability of trajectory class membership > 70%

|  | | All  patients | Univariate analyses | | | | Multinomial logistic regression analysis | | |
| --- | --- | --- | --- | --- | --- | --- | --- | --- | --- |
|  |  |  | Class 1 | Class 2 | Class 3 | *p*-value^a^ | OR (95% CI) | | |
|  |  |  |  |  |  |  | Class 2^b^ | Class 3^b^ | *p*-value^a^ |
| Number | | 187 | 31 (16.6) | 89 (47.6) | 67 (35.8) |  |  |  |  |
| Female | | 34 (18.2) | 4 (12.9) | 15 (16.9) | 15 (22.4) | 0.476 |  |  |  |
| Age at diagnosis, years | | 25.5 (9.6) | 23.6 (8.5) | 24.5 (9.3) | 27.7 (10.5) | **0.062** | 1.015  (0.965–1.069) | 1.044  (0.991–1.099) | 0.150 |
| Current smoker | | 25 (13.4) | 4 (12.9) | 10 (11.2) | 11 (16.4) | 0.640 |  |  |  |
| History of appendectomy | | 14 (7.5) | 1 (3.2) | 6 (6.7) | 7 (10.4) | 0.421 |  |  |  |
| CCI | 0 | 177 (94.7) | 30 (96.8) | 86 (96.6) | 61 (91.0) | 0.484 |  |  |  |
|  | 1 | 9 (4.8) | 1 (3.2) | 3 (3.4) | 5 (7.5) |  |  |  |  |
|  | ≥ 2 | 1 (0.5) | 0 (0.0) | 0 (0.0) | 1 (1.5) |  |  |  |  |
| Positive ANCA titer | | 2 (2.1) | 0 (0.0) | 1 (2.4) | 1 (2.9) | 0.759 |  |  |  |
| Elevation of ASCA IgG or IgA | | 114 (61.3) | 16 (51.6) | 55 (62.5) | 43 (64.2) | 0.469 |  |  |  |
| Disease location | Ileal | 43 (23.0) | 7 (22.6) | 19 (21.3) | 17 (25.4) | 0.526 |  |  |  |
|  | Colonic | 12 (6.4) | 4 (12.9) | 4 (4.5) | 4 (6.0) |  |  |  |  |
|  | Ileocolonic | 132 (70.6) | 20 (64.5) | 66 (74.2) | 46 (68.7) |  |  |  |  |
| UGI involvement | | 24 (12.8) | 3 (9.7) | 8 (9.0) | 13 (19.4) | **0.133** | 1.090  (0.251–4.739) | 2.335  (0.554–9.837) | 0.300 |
| Disease behavior | Nonstricturing, nonpenetrating | 120 (64.2) | 22 (71.0) | 59 (66.3) | 39 (58.2) | **0.081** |  |  | 0.300 |
|  | Stricturing | 25 (13.4) | 1 (3.2) | 9 (10.1) | 15 (22.4) |  | 1.822  (0.201–16.53) | 4.671  (0.534–40.88) |  |
|  | Penetrating | 42 (22.5) | 8 (25.8) | 21 (23.6) | 13 (19.4) |  | 0.789  (0.271–2.301) | 0.846  (0.269–2.660) |  |
| Perianal disease | | 91 (48.7) | 17 (54.8) | 40 (44.9) | 34 (50.7) | 0.582 |  |  |  |
| Early CS | | 120 (64.2) | 18 (58.1) | 64 (71.9) | 38 (56.7) | **0.109** | 2.237  (0.821–6.094) | 1.341  (0.470–3.827) | 0.200 |
| Early IM | | 156 (83.4) | 27 (87.1) | 76 (85.4) | 53 (79.1) | 0.483 |  |  |  |
| Early AT | | 51 (27.3) | 14 (45.2) | 24 (27.0) | 13 (19.4) | **0.029** | 0.251  (0.095–0.669) | 0.206  (0.069–0.614) | **0.008** |
| Early intestinal resection | | 5 (2.7) | 0 (0.0) | 3 (3.4) | 2 (3.0) | 0.594 |  |  |  |
| Early ER visit^c^ | | 28 (15.0) | 0 (0.0) | 17 (19.1) | 11 (16.4) | **0.034** |  |  | **0.023** |
| Early hospitalization | | 34 (18.2) | 2 (6.5) | 21 (23.6) | 11 (16.4) | **0.093** | 1.925  (0.359–10.31) | 1.404  (0.224–8.777) | 0.700 |

| CCI, Charlson Comorbidity Index; ANCA, anti-neutrophil cytoplasmic antibody; ASCA, anti-*Saccharomyces cerevisiae* antibody; CS, corticosteroid, IM, immunomodulator; AT, advanced therapy; ER, emergency room  Values are presented as the mean (standard deviation) or number (%).  ^a^ *p*-value is indicated in bold when < 0.200 in univariate analyses and when < 0.050 in multinomial logistic regression analysis.  ^b^ All groups were compared with class 1.  ^c^ Comparisons were not available because no patients in class 1 had a history of early ER visits. |
| --- |

**Supplementary Table 4.** Clinical characteristics, univariate analyses, and multinomial logistic regression analysis based on longitudinal trajectories of C-reactive protein in patients with ulcerative colitis with a probability of trajectory class membership > 70%

|  | | Total patients | Univariate analyses | | | | Multinomial logistic regression analysis | | |
| --- | --- | --- | --- | --- | --- | --- | --- | --- | --- |
|  |  |  | Class 1 | Class 2 | Class 3 | *p*-value^a^ | OR (95% CI) | | *p*-value^a^ |
|  |  |  |  |  |  |  | Class 2^b^ | Class 3^b^ |  |
| Number | | 581 | 528 (90.9) | 38 (6.5) | 15 (2.6) |  |  |  |  |
| Female | | 217 (37.3) | 200 (37.9) | 12 (31.6) | 5 (33.3) | 0.702 |  |  |  |
| Age at diagnosis, years | | 37.9 (14.2) | 37.5 (13.8) | 44.3 (20.2) | 34.3 (12.0) | **0.011** | 1.026  (0.998–1.054) | 1.027  (0.989–1.067) | 0.094 |
| Current smoker | | 90 (15.5) | 83 (15.7) | 3 (7.9) | 4 (26.7) | 0.209 |  |  |  |
| History of appendectomy | | 16 (2.8) | 14 (2.7) | 2 (5.3) | 0 (0.0) | 0.512 |  |  |  |
| CCI | 0 | 484 (83.3) | 441 (83.5) | 30 (78.9) | 13 (86.7) | 0.882 |  |  |  |
|  | 1 | 67 (11.5) | 61 (11.6) | 5 (13.2) | 1 (6.7) |  |  |  |  |
|  | ≥ 2 | 30 (5.2) | 26 (4.9) | 3 (7.9) | 1 (6.7) |  |  |  |  |
| Positive ANCA titer | | 53 (14.1) | 41 (12.1) | 9 (36.0) | 3 (25.0) | **0.002** | 2.918  (1.185–7.187) | 0.858  (0.204–3.604) | 0.073 |
| Elevation of ASCA IgG or IgA | | 92 (17.8) | 80 (16.9) | 8 (25.8) | 4 (30.8) | 0.211 |  |  |  |
| Disease extent | Proctitis | 227 (39.1) | 214 (40.5) | 10 (26.3) | 3 (20.0) | **0.018** |  |  | 0.400 |
|  | Left-sided colitis | 168 (28.9) | 154 (29.2) | 12 (31.6) | 2 (13.3) |  | 1.334  (0.472–3.770) | 0.517  (0.075–3.557) |  |
|  | Extensive colitis | 186 (32.0) | 160 (30.3) | 16 (42.1) | 10 (66.7) |  | 1.425  (0.482–4.216) | 1.958  (0.427–8.975) |  |
| Early CS | | 182 (31.3) | 158 (29.9) | 14 (36.8) | 10 (66.7) | **0.008** | 0.897  (0.330–2.441) | 2.111  (0.536–8.315) | 0.500 |
| Early IM | | 132 (22.7) | 112 (21.2) | 10 (26.3) | 10 (66.7) | **< 0.001** | 0.830  (0.302–2.280) | 3.610  (1.120–11.64) | 0.085 |
| Early AT | | 10 (1.7) | 3 (0.6) | 5 (13.2) | 2 (13.3) | **< 0.001** | 9.759  (1.024–92.99) | 4.865  (0.488–48.46) | 0.110 |
| Early ER visit | | 37 (6.4) | 28 (5.3) | 5 (13.2) | 4 (26.7) | **0.001** | 0.991  (0.150–6.563) | 1.537  (0.175–13.50) | >0.999 |
| Early hospitalization | | 32 (5.5) | 22 (4.2) | 6 (15.8) | 4 (26.7) | **< 0.001** | 4.099  (0.678–24.78) | 2.634  (0.276–25.18) | 0.300 |

| CCI, Charlson Comorbidity Index; ANCA, anti-neutrophil cytoplasmic antibody; ASCA, anti-*Saccharomyces cerevisiae* antibody; CS, corticosteroid, IM, immunomodulator; AT, advanced therapy; ER, emergency room |
| --- |
| Values are presented as the mean (standard deviation) or number (%).  ^a^ *p*-value is indicated in bold when < 0.200 in univariate analyses and when < 0.050 in multinomial logistic regression analysis.  ^b^ All groups were compared with class 1. |

**Supplementary Table 5.** Clinical characteristics, univariate analyses, and multinomial logistic regression analysis based on longitudinal trajectories of fecal calprotectin in patients with ulcerative colitis with a probability of trajectory class membership > 70%

|  | | Total patients^a^ | Univariate analyses | | | | Multinomial logistic regression analysis | | |
| --- | --- | --- | --- | --- | --- | --- | --- | --- | --- |
|  |  |  | Class 1^a^ | Class 2^a^ | Class 3^a^ | *p*-value^b^ | OR (95% CI) | | *p*-value^b^ |
|  |  |  |  |  |  |  | Class 2^c^ | Class 3^c^ |  |
| Number | | 374 | 176 (47.1) | 22 (5.9) | 176 (47.1) |  |  |  |  |
| Female | | 140 (37.4) | 69 (39.2) | 9 (40.9) | 62 (35.2) | 0.699 |  |  |  |
| Age at diagnosis, years | | 38.6 (14.1) | 41.0 (12.5) | 37.5 (17.0) | 36.4 (15.4) | **0.010** | 0.971  (0.938–1.005) | 0.977  (0.962–0.993) | **0.010** |
| Current smoker^d^ | | 54 (14.4) | 34 (19.3) | 0 (0.0) | 20 (11.4) | **0.015** |  | 0.430  (0.227–0.817) | **< 0.001** |
| History of appendectomy | | 10 (2.7) | 5 (2.8) | 0 (0.0) | 5 (2.8) | 0.725 |  |  |  |
| CCI | 0 | 306 (81.8) | 145 (82.4) | 20 (90.9) | 141 (80.1) | 0.378 |  |  |  |
|  | 1 | 47 (12.6) | 23 (13.1) | 0 (0.0) | 24 (13.6) |  |  |  |  |
|  | ≥ 2 | 21 (5.6) | 8 (4.5) | 2 (9.1) | 11 (6.3) |  |  |  |  |
| Positive ANCA titer | | 44 (16.7) | 18 (16.7) | 3 (18.8) | 23 (20.2) | 0.797 |  |  |  |
| Elevation of ASCA IgG or IgA | | 56 (14.3) | 23 (14.3) | 3 (15.0) | 30 (19.2) | 0.487 |  |  |  |
| Disease extent | Proctitis | 136 (36.4) | 69 (39.2) | 10 (45.5) | 57 (32.4) | **0.108** |  |  | 0.089 |
|  | Left-sided colitis | 107 (28.6) | 50 (28.4) | 9 (40.9) | 48 (27.3) |  | 0.583  (0.189–1.797) | 0.802  (0.450–1.431) |  |
|  | Extensive colitis | 131 (35.0) | 57 (32.4) | 3 (13.6) | 71 (40.3) |  | 0.150  (0.032–0.704) | 0.902  (0.502–1.619) |  |
| Early CS | | 130 (34.8) | 50 (28.4) | 10 (45.5) | 70 (39.8) | **0.045** | 3.048  (1.037–8.953) | 1.131  (0.659–1.941) | 0.130 |
| Early IM | | 100 (26.7) | 26 (14.8) | 8 (36.4) | 66 (37.5) | **< 0.001** | 3.461  (1.178–10.17) | 3.581  (2.052–6.247) | **< 0.001** |
| Early AT | | 11 (2.9) | 3 (1.7) | 1 (4.5) | 7 (4.0) | 0.406 |  |  |  |
| Early ER visit | | 30 (8.0) | 13 (7.4) | 0 (0.0) | 17 (9.7) | 0.265 |  |  |  |
| Early hospitalization^d^ | | 26 (7.0) | 9 (5.1) | 0 (0.0) | 17 (9.7) | **0.102** |  | 1.528  (0.590–3.958) | 0.200 |

| CCI, Charlson Comorbidity Index; ANCA, anti-neutrophil cytoplasmic antibody; ASCA, anti-*Saccharomyces cerevisiae* antibody; CS, corticosteroid, IM, immunomodulator; AT, advanced therapy; ER, emergency room |
| --- |
| Values are presented as the mean (standard deviation) or number (%).  ^a^ *p*-value is indicated in bold when < 0.200 in univariate analyses and when < 0.050 in multinomial logistic regression analysis.  ^b^ All groups were compared with class 1.  ^d^ Classes 1 and 2 could not be compared because no patients in class 2 were current smokers or had a history of early hospitalization. |

**Supplementary Table 6.** Comparison of ER visit, hospitalization, and intestinal resection proportions according to CRP and FCP trajectories in patients with CD and UC.

|  | | **ER visit** | | **Hospitalization** | | **Intestinal resection** | |
| --- | --- | --- | --- | --- | --- | --- | --- |
|  |  | **N (%)** | ***p*-value** | **N (%)** | ***p*-value** | **N (%)** | ***p*-value** |
| **CD-CRP** | Class 1 | 32 (23.5) | 0.039 | 31 (22.8) | 0.005 | 6 (4.5) | 0.410 |
|  | Class 2 | 9 (37.5) |  | 8 (33.3) |  | 2 (8.3) |  |
|  | Class 3 | 33 (38.8) |  | 37 (43.5) |  | 12 (14.1) |  |
| **CD-FCP** | Class 1 | 4 (8.5) | 0.001 | 5 (10.6) | 0.002 | 1 (2.1) | 0.246 |
|  | Class 2 | 41 (33.9) |  | 39 (32.2) |  | 12 (9.9) |  |
|  | Class 3 | 33 (37.5) |  | 35 (39.8) |  | 7 (8.0) |  |
| **UC-CRP** | Class 1 | 73 (12.9) | <0.001 | 63 (11.2) | <0.001 | 4 (0.7) | 0.200 |
|  | Class 2 | 25 (50.0) |  | 26 (52.0) |  | 3 (6.0) |  |
|  | Class 3 | 7 (33.3) |  | 7 (33.3) |  | 0 (0.0) |  |
| **UC-FCP** | Class 1 | 31 (12.8) | 0.009 | 24 (9.9) | 0.002 | 0 (0.0) | 0.054 |
|  | Class 2 | 13 (31.0) |  | 10 (23.8) |  | 1 (2.4) |  |
|  | Class 3 | 47 (18.7) |  | 51 (20.3) |  | 5 (2.0) |  |

CD, Crohn’s disease; CRP, C-reactive protein; ER, emergency room; FCP, fecal calprotectin; N, number; UC, ulcerative colitis
