# Supplementary figures and images for: Impact of early aggressive treatment on long-term biochemical marker patterns in inflammatory bowel disease
Source: J Gastroenterol. 2025 May 2;60(7):854–65. doi: 10.1007/s00535-025-02244-w (PMC12176982; doi:10.1007/s00535-025-02244-w)

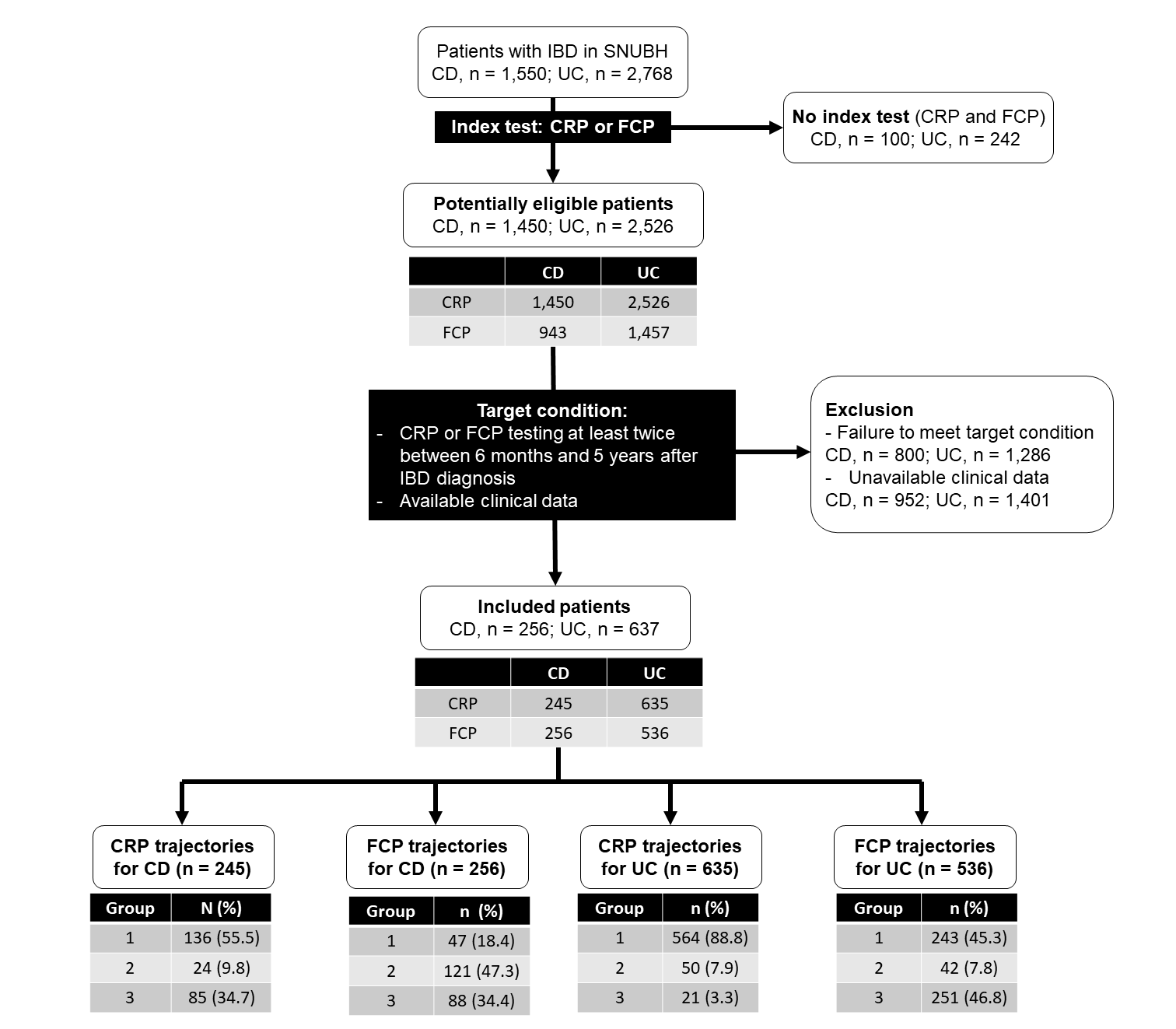

Supplement: Supplementary file 2 — Supplementary file2 (TIF 244 KB) Supplementary Fig. 1. Standards for Reporting of Diagnostic Accuracy (STARD) diagram for the study. The flow diagram illustrates the recruitment and classification of study participants according to the STARD guideline [file 535_2025_2244_MOESM2_ESM.tif]

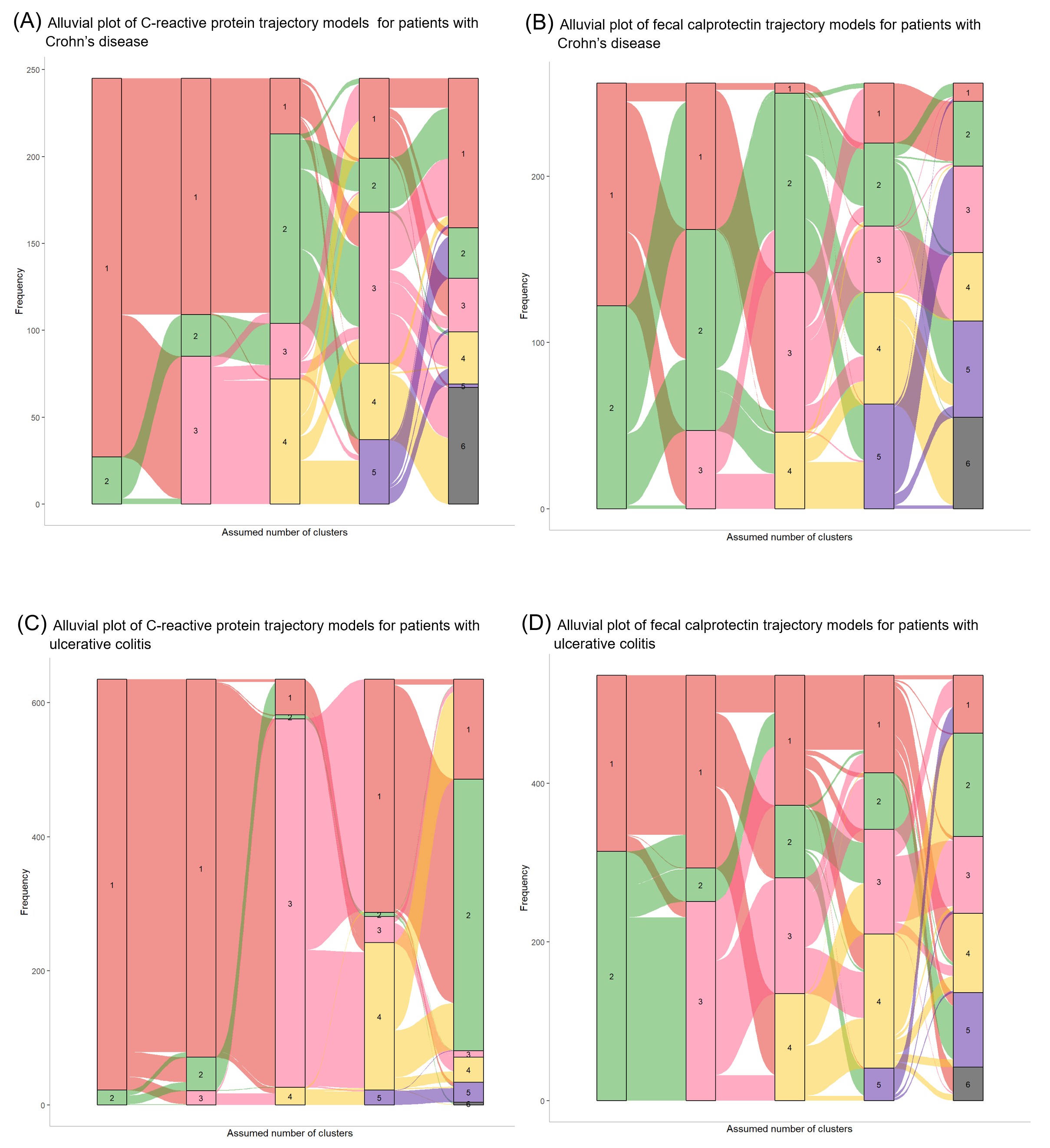

Supplement: Supplementary file 3 — Supplementary file3 (TIF 2285 KB) Supplementary Fig. 2. Alluvial plots show how the distribution of class membership among patients changed with varying numbers of C-reactive protein or fecal calprotectin trajectory classes. (A) Alluvial plot of C-reactive protein (CRP) trajectory models for patients with Crohn’s disease (CD). (B) Alluvial plot of fecal calprotectin (FCP) trajectory models for patients with CD. (C) Alluvial plot of CRP trajectory models for patients with ulcerative colitis (UC). (D) Alluvial plot of FCP trajectory models for patients with UC [file 535_2025_2244_MOESM3_ESM.tif]

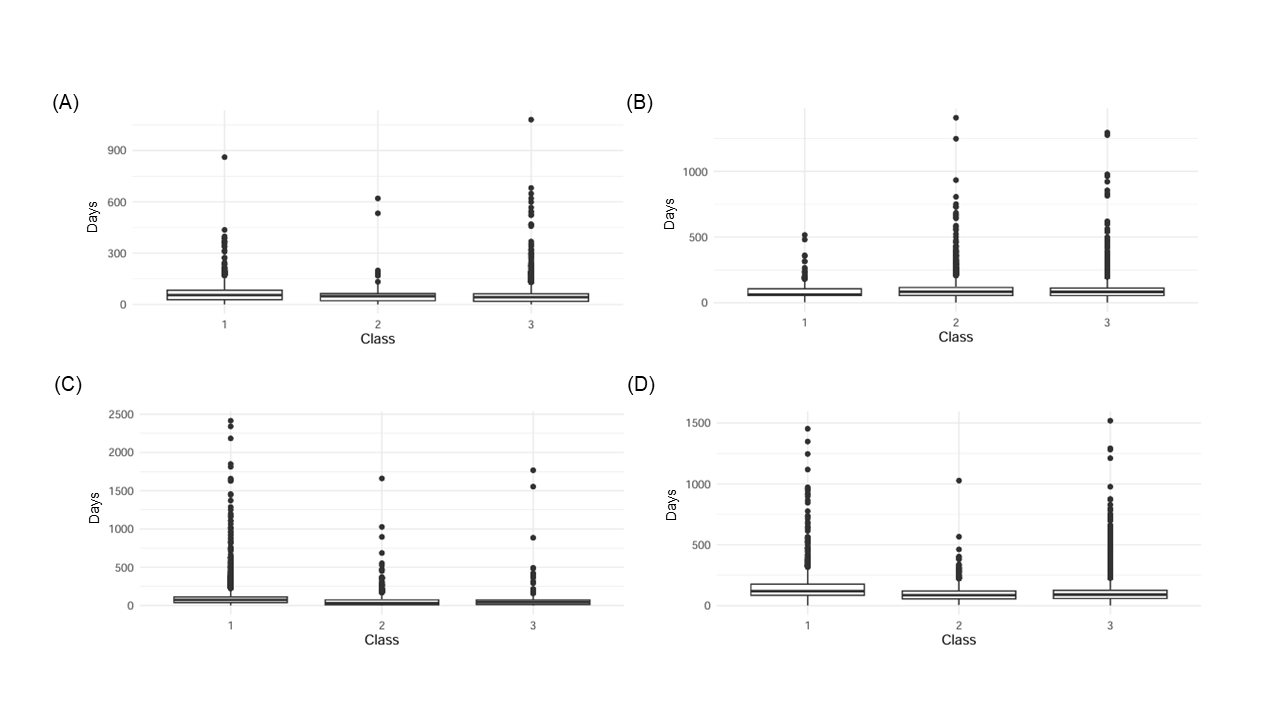

Supplement: Supplementary file 4 — Supplementary file4 (TIF 91 KB) Supplementary Fig. 3. Comparison of testing intervals across classes using box plots. The bottom and top edges of the box represent the first (Q1) and third quartiles (Q3), indicating the 25 th and 75 th percentiles of the data, respectively. The line inside the box represents the median (Q2), which corresponds to the 50 th percentile. The whisker extends from the bottom or top of the box to the smallest or largest data point, which is 1.5 times the interquartile range from Q1 or Q3. Individual points outside the whiskers are considered outliers. Although outliers are present, the position and size of the boxes do not differ noticeably across classes. (A) A box plot of C-reactive protein (CRP) testing intervals in patients with Crohn’s disease (CD). (B) A box plot of fecal calprotectin (FCP) testing intervals in patients with CD. (C) A box plot of CRP testing intervals in patients with ulcerative colitis (UC). (D) A box plot of FCP testing intervals in patients with UC [file 535_2025_2244_MOESM4_ESM.tif]
